# The dental demolition derby: bruxism and its impact - part 3: repair and reconstruction

**DOI:** 10.1038/s41415-022-4293-8

**Published:** 2022-06-10

**Authors:** Mark. L. T. Thayer, Rahat Ali

**Affiliations:** 41415113454001 Consultant and Honorary Lecturer in Oral Surgery, Liverpool University Dental Hospital, Pembroke Place, Liverpool, L3 5PS, UK; 41415113454002 Consultant in Restorative Dentistry, Liverpool University Dental Hospital, Pembroke Place, Liverpool, L3 5PS, UK

## Abstract

Bruxism is a term that encompasses a range of presentations of rhythmic and repetitive muscular activity. For many, this is not a significant problem but for some, this behaviour leads to substantial impact and tissue damage that can be significant, compromising function and quality of life. This paper will review management methods for reconstructing the damaged dentition.

## Key points

- Reconstruction is complex. Interventions should be pragmatic and guidance is provided in a themed approach.
- Interventions may be conformative or reconstructive. The grinding style should be identified to allow reconstruction to work with activity, not in conflict.
- Preservation of tooth tissue is essential and direct placed bonded composite materials are biologically friendly, easily repaired, cheap and act as stress breakers.
- Complex fixed restorative reconstruction and implant supported bridges are likely to have high failure rates and must be protected with occlusal splints. Reconstructions should be planned with maximum retrievability and removable options figure significantly in effective treatment plans.

## Introduction

**Fig. 1:**
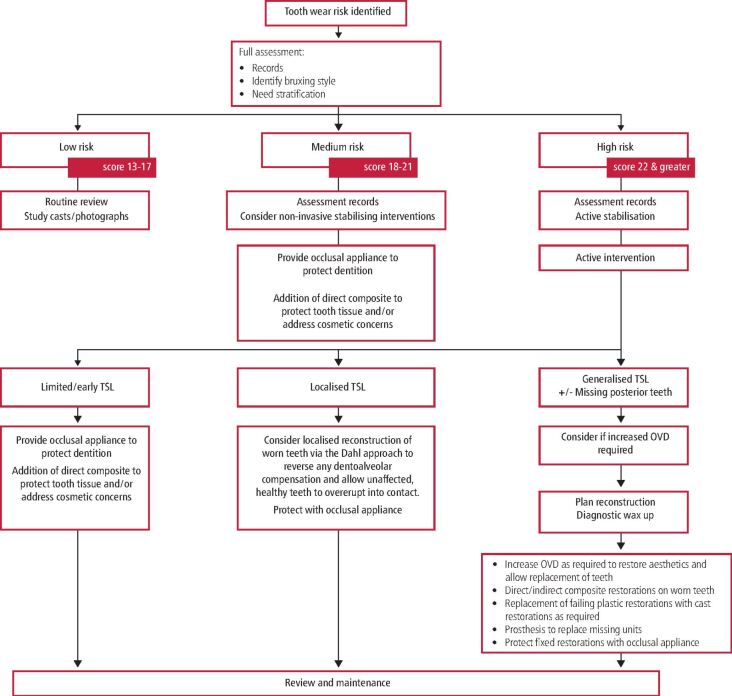
Flow diagram demonstrating application of intervention need matrix and thematic management of cases depending upon severity of need. For this paper, patients will be presenting with scores greater than 21

In the second part of this series, the authors discussed methods for controlling bruxing to limit its destructive impact on the dentition. However, when extensive destruction of the dentition by bruxism has occurred, reconstruction will be required. In the context of the treatment need stratification matrix introduced by the authors in the first paper, such patients would be expected to score greater than 22, indicating a high likelihood (or need) for intervention. It is important that the reader should appreciate that there are no specific ideal treatment plans, and approaches are largely pragmatic and thematic. Themes are summarised in the intervention flow diagram (Fig. 1) and in the context of this paper, interventions will be largely based on the theme of the right section of the flow diagram.

Reconstruction must take into account the hostile environment and requires choices to maximise longevity of the intervention, but an essential component of any plan is to include built-in options for rescue of any failure. The concept of a single definitive 'fix' for the extensively damaged dentition is commonly sought. This may not be feasible given the hostile functional environment that is produced during bruxing. This should be made clear to the patient as part of their consent process before embarking on treatment. It forms an integral part of their rehabilitation.

In this third and final paper, the authors will discuss various strategies that can be used to restore an extensively damaged dentition that has occurred because of bruxism.

## Planning the reconstruction phase

The dental reconstruction of a patient presenting with extensive tooth wear in the context of destructive bruxism is very difficult. The increased occlusal forces,^1^^,^^2^ non-axial loading and increased length of time the restorations are under heavier occlusal load all contribute to a shorter period of time between restoration placement and intervention to repair or replace. Studies have shown a higher failure rate of zirconia crowns^3^ in patients with a parafunctional habit. Even removable overlay/onlay dentures are not without their problems in a bruxing patient; Woodley *et al*.^4^ noticed 64% of their tooth wear patients had problems with their removable onlay/overlay dentures and included issues such as wear of the acrylic and tooth fracture. This is not surprising, given that patients with a bruxing habit were also included in their audit.

Diagnosis is the key to successful treatment planning, along with pragmatic decision-making, supported by the need stratification matrix when appropriate. However, a number of other factors do require consideration:

1. The nature and 'style' of bruxing. In this cohort of patients, bruxing activity is the primary issue leading to the tooth wear. If possible, the nature of bruxing should be identified. This is difficult and may include lateral (guided) grinding, anterior grinding posturing onto the anterior teeth, canine grinding or clenching, simple (static or isometric) clenching, rhythmic clenching, exploratory grinding and soft tissue 'play' (for example, lip nibbling). If it is accepted that the drive for bruxing is centrally placed, then identification of the type of activity will help to plan the reconstruction, as the reconstruction can be planned to be sympathetic to the behaviour, rather than create conflict. Provision of an interim occlusal appliance to act as a diagnostic tool to examine the bruxing behaviour can be an integral component of the first stages of reconstruction
2. Medical comorbidities must influence the decision-making process, as these may predispose to significant complications. For example, cancer patients taking intravenous bisphosphonates may have limited surgical options available to them for the reconstructive phase of their rehabilitation. The authors would strongly counsel against implant placement in this patient cohort as they would be at a higher risk of osteonecrosis^5^^,^^6^^,^^7^^,^^8^
3. Other conditions may complicate provision of complex reconstruction: movement disorders, stroke, or other neurological or physical conditions may make extensive reconstruction work difficult and may prevent effective patient maintenance. Trismus may limit access and patient reflexes, such as the gag reflex, may prevent even, effective examination
4. Anxiety and tolerance should also be considered - is the patient able to tolerate extensive reconstruction and the time input it requires? A partially reconstructed situation is worse than no intervention and a reconstruction that is impossible for the patient to maintain will catastrophically fail
5. Periodontal disease - all active gingivitis and periodontitis must have been treated and be under control. Patients should demonstrate excellent plaque control on multiple appointments before considering complex reconstructive therapy
6. A good principle to keep in mind during planning of reconstruction is 'to keep it simple'. A relatively simple restorative reconstruction should be relatively simple and straightforward to maintain when failure of the treatment inevitably occurs
7. What is left to work with? Is there sufficient tooth structure to use direct/indirect adhesive restorations? Or are simply roots remaining that may commit the patient to a removable overdenture?

**Fig. 2:**
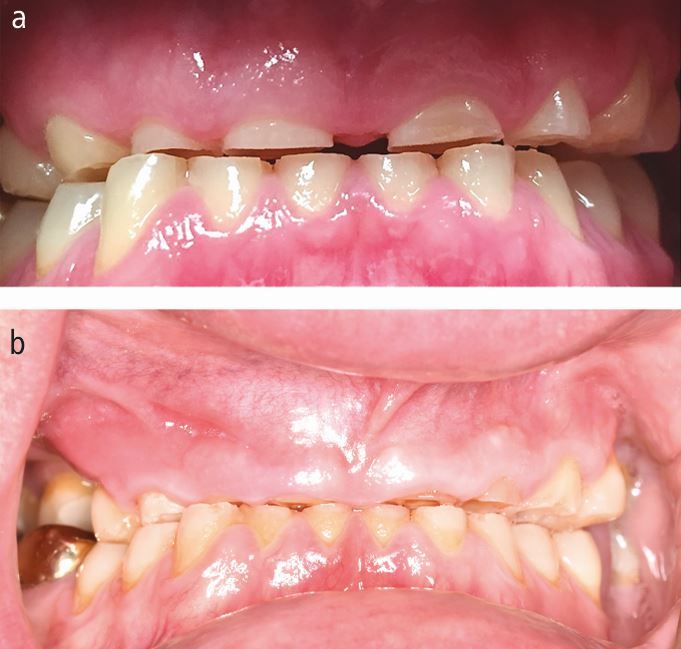
a) Patient at age 23; b) Same patient at 29 years of age who continued to grind his teeth and drink carbonated drinks

When the dentition is severely worn by bruxing, reconstruction of the teeth will be challenging. A primary issue lies in the lack of clinical crown height and some patients have no crown at all, having worn their teeth to gingival level (Figures 2a and 2b).

Tooth structure should be considered sacrosanct, as this has already been compromised by the destructive nature of the parafunctional environment. Extensive crown preparations on such teeth can compromise tooth structure as well as pulpal survival due to them possibly having a 'stressed pulp' from the pre-existing attrition.^9^ Neither enamel nor pulp can be replaced, committing the tooth to long-term cycles of ever increasingly destructive reconstruction which may, over time, hasten its loss. Therefore, attempts at reconstruction of the coronal tissue with full coverage crowns may be inappropriate in a severely worn dentition. The preparation alone may reduce the retention and resistance form of the crown, increasing the risk of failure from the outset. To offset this issue, crown lengthening surgery (to increase the retention form for the proposed crown) could be considered. However, the high occlusal loads can cause the crowns to fracture and the teeth to mobilise should the crown-to-root ratio (following bone removal) be unfavourable and is overwhelmed by the occlusal load. Smales and Berekally^10^ examined the prescription of composite or crowns to manage a small proportion of tooth wear patients. Tooth grinding was thought to be the main cause for tooth wear in many of their patients. They noticed that there was no statistically significant difference in failure rate between the use of composite resin or indirect crowns to treat their patients. However composite failure was often simple to repair or replace, while crown failures often committed the patient to either root canal therapy or extraction. Although their case series only examined 25 patients, prescription of crowns in tooth wear patients (especially with a parafunctional habit) must be considered with caution. In such a hostile environment, prescription of readily repairable composite resin in moderately worn cases or an overdenture for more severe cases may be more reasonable and conservative than crown provision, with or without crown lengthening surgery.

The concepts of enamel conservation outlined by Kelleher^11^^,^^12^ should be a vital point of reference. Composite-based reconstruction supports this conservative approach. Although adhesively bonded restorations are likely to fracture over time, they represent a purely additive approach to manage a worn tooth. Unlike a crown preparation, a composite restoration is unlikely to cause a worn tooth to decoronate. Furthermore, it can be repaired as an ongoing process.^12^^,^^13^^,^^14^

## Localised or generalised reconstruction of the damaged dentition?

During reconstruction, planning will broadly break down into two groups: those with localised tooth wear and those with extensive tissue loss surface loss.

### Localised tooth wear

**Fig. 3:**
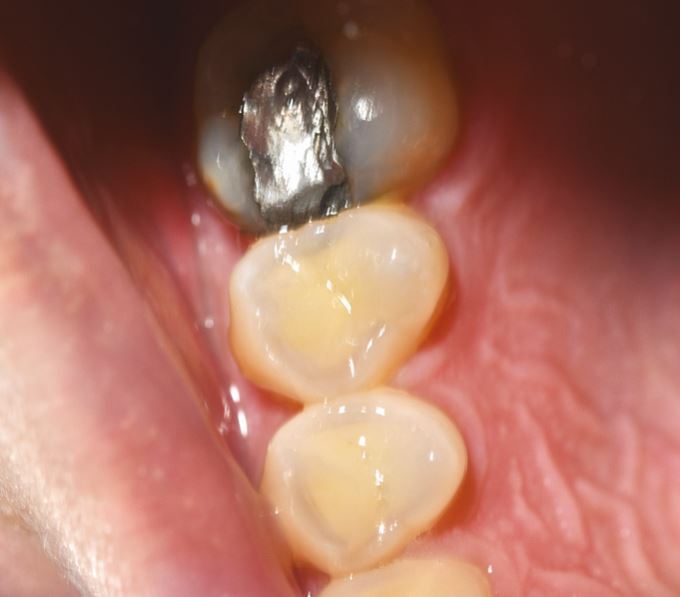
Selective (specific grinding style) wear of 14 and 15 buccal cusps in 25-year-old bruxist with intervention score of 25. Note superficial appearance of erosion is not confirmed when compared to 16 which shows no damage

**Fig. 4:**
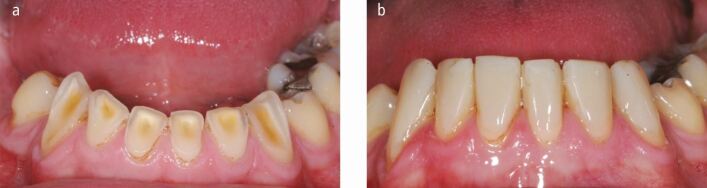
a, b) A 24-year-old nocturnal bruxist patient who drank diet cola. During the day at work he (and his partner) reported that he would posture his jaw forwards slide his teeth forwards and backwards, accelerating his already worn lower dentition

This is likely to represent a localised grinding style, with specific posturing of the mandible to one side or to the anterior teeth (Figures 3 and 4). In such cases, the repair of the teeth will generally be localised. A decision to undertake entire mouth rehabilitation is generally not necessary and in such cases, a loss of vertical dimension is generally not an issue due to dentoalveolar compensation.

Repair of teeth with localised wear may be managed with composite restorations. The occlusal scheme should be designed to ensure that there are bilateral, even contacts in the patient's retruded contact position, as well as the jaw relationship where they have postured and are grinding. This is typically on the anterior teeth and therefore, there should be shared guidance in protrusive mandibular movements. If posterior teeth are being restored, they should be contoured to ensure that there are no working side or non-working side interferences. If there has been dentoalveolar compensation, the restorations can be placed at an increased occlusal vertical dimension to restore aesthetics and ensure that the material used is durable in cross section. The remaining occlusal contacts will re-establish due to the relative axial tooth movement that will occur when localised restorations are provided at an increased vertical dimension and the teeth will typically re-establish positions over 6-9 months, as per the well-described 'Dahl concept'.^15^

#### The Dahl appliance

**Fig. 5:**
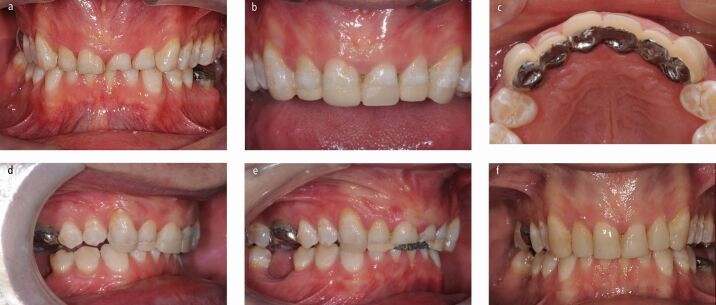
a, b, c, d, e, f) A severe bruxing patient who would posture forwards and grind down his anterior teeth. Previous attempts had been made to build up his worn maxillary incisor and canine teeth with composite resin at an increased OVD. However, he fractured these restorations off within a few weeks before the posterior teeth re-established their occlusal contacts. A fixed Dahl Appliance with a non-precious metal bite platform palatally and acrylic teeth anteriorly was cemented on with glass ionomer cement to create anterior inter occlusal space. After 6 months, the appliance was removed and the resulting anterior open bite was restored with composite resin at the new increased vertical dimension

While the localised worn dentition can be restored in supra-occlusion and the remaining dentition allowed re-establish contact with their antagonistic teeth (Figures 4a and 4b), this approach may prove more troublesome in a select group of patients with a severe parafunctional habit with a tendency to fracture any restorations that are placed at an increased vertical dimension (for example incisal edge repairs). Such patients may benefit from orthodontic therapy to create interocclusal space to allow restoration of the worn teeth, but this would take time, may be socially unacceptable and may be difficult to achieve in a mature patient. It would bring technical challenges, such as bonding brackets to teeth with an already short clinical crown height. In such patients, a classical, fixed Dahl appliance may be useful to create the interocclusal space required to restore any worn teeth (Figures 5a, 5b, 5c, 5d, 5e and 5f).

### Extensive tooth wear

When presented with extensive tooth wear, loss of vertical facial height may become an issue if the rate of tooth wear overwhelms the dentoalveolar compensatory mechanisms. In general, this does not seem to be the case as tooth wear tends to be a gradual process. Since nature compensates to maintain the vertical dimension, there is often no decrease in the vertical dimension, even for more severe types of tooth wear.^16^ If vertical height has been lost, it may relate to rapid forms of tooth wear, or alternatively, to dentitions where patients who are already missing most of their posterior teeth and are now exclusively (para)functioning on their remaining anterior teeth. The rate of tissue loss is increased, simply because the workload is no longer distributed across a full dentition.

**Fig. 6:**
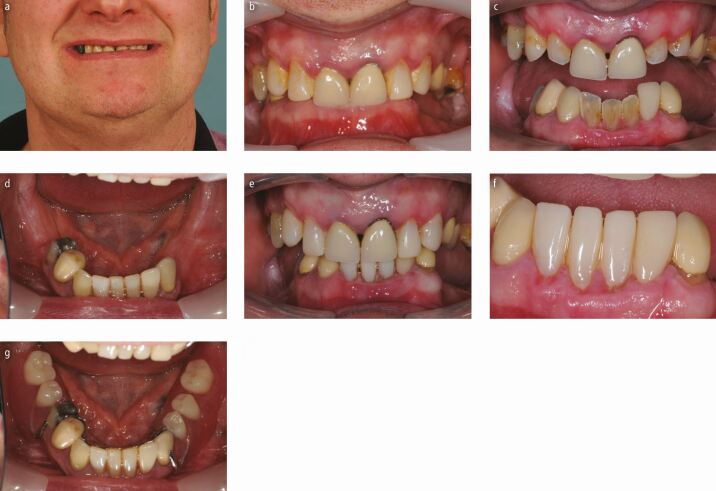
a, b, c, d, e, f, g) A 41-year-old patient who was aware of grinding his teeth during the day when stressed. He was struggling to chew as he was missing so many teeth and was concerned about the wear on his remaining anterior dentition. Notice the labial periodontal trauma around his mandibular anterior teeth and his slightly reduced face height. He felt that over the years, his 'nose was getting closer to his chin'. The lower worn incisors were restored with composite at an increased vertical dimension to prevent further periodontal trauma and restore aesthetics. The posterior teeth were replaced with a partial chrome denture to increase posterior occlusal support. A lingual plate connector was prescribed to allow easy future addition should any of the remaining worn teeth require extraction in the future

**Fig. 7:**
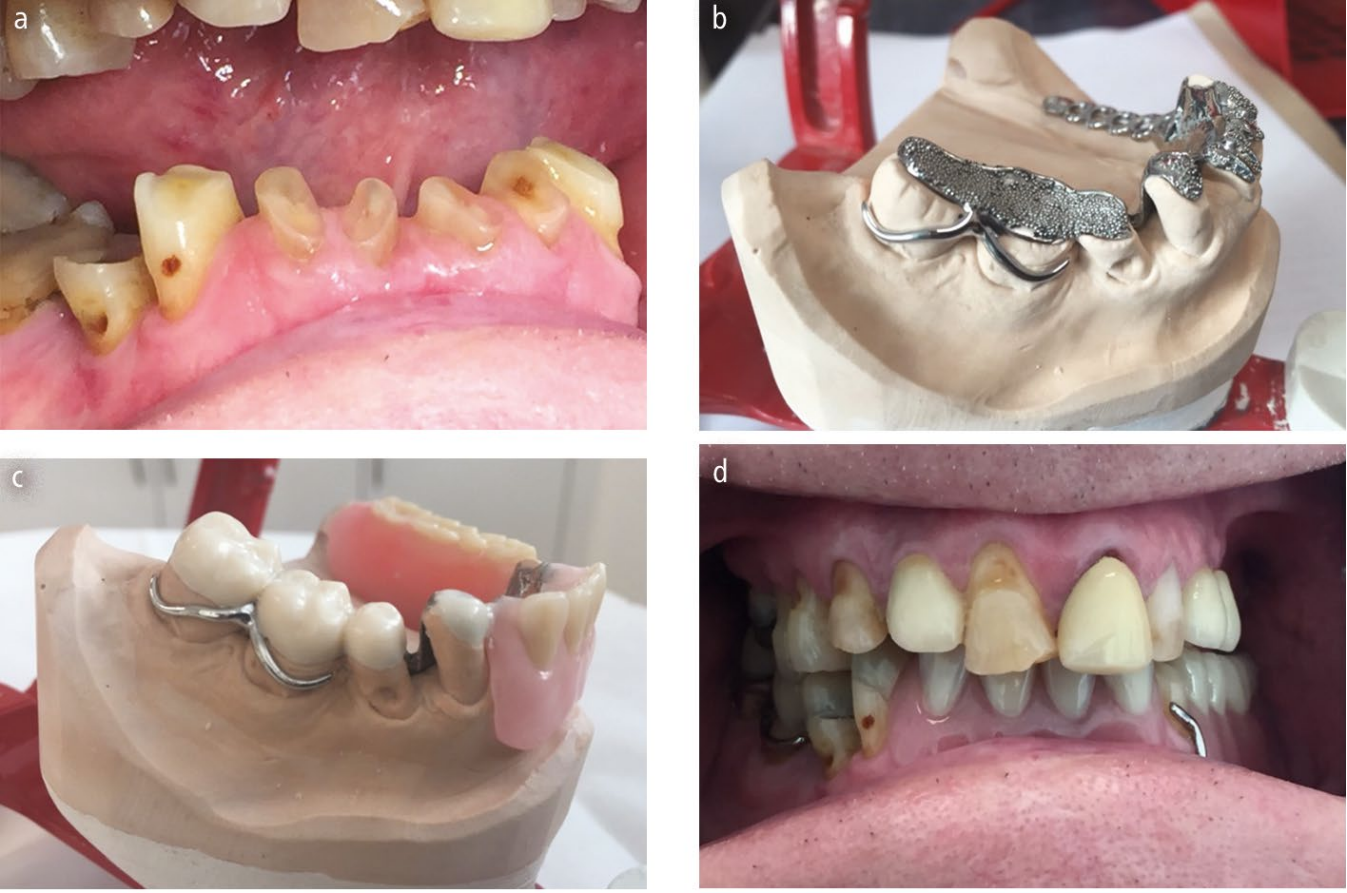
a, b, c, d) A bruxist missing a number of posterior teeth. His remaining teeth were previously restored with composite resin at an increased OVD whilst the missing posterior teeth were replaced with a partial denture at the increased vertical dimension. The composite restorations failed every few months and subsequently he stopped wearing his partial denture. He was rehabilitated with a mandibular, partial, chrome-based overlay/onlay denture. The worn anterior teeth were overlayed with acrylic teeth and any missing posterior units were also replaced. Given that the prosthesis was made at an increased OVD, the remaining posterior teeth on the right side were onlayed to bring them back into contact and control the occlusal scheme. Chrome beads were used to retain acrylic teeth but ideally metal occlusal surfaces should have been prescribed for this case as it will fail at a much slower rate than acrylic

In such severe cases, the occluding vertical dimension (OVD) should be increased to allow reconstruction of the worn teeth so that the restorations are both durable in function and aesthetically pleasing. In clinical practice, a balance of these two factors is required during the planning phases. Reconstruction would normally involve rebuilding any restorable teeth with fixed restorations at the increased OVD and replacing any missing posterior units to provide posterior occlusal support at this OVD to limit parafunctional forces on the remaining fixed restorations,^17^ effectively spreading the loading, as well as to limit any further tooth wear on the anterior teeth.^18^ Figure 6 is an example of such a treatment plan. If, however, the remaining teeth have an unacceptably high failure rate of fixed restorations placed on them, then an alternative approach may be to replace any missing posterior units and to overlay any severely worn anterior teeth at the new OVD (Figure 7). This would certainly decrease the maintenance burden of any fixed restorations, but the patient would need regular 3-4-monthly dental follow-up appointments to ensure they are maintaining optimal plaque control around the overlayed and abutment teeth.

**Fig. 8:**
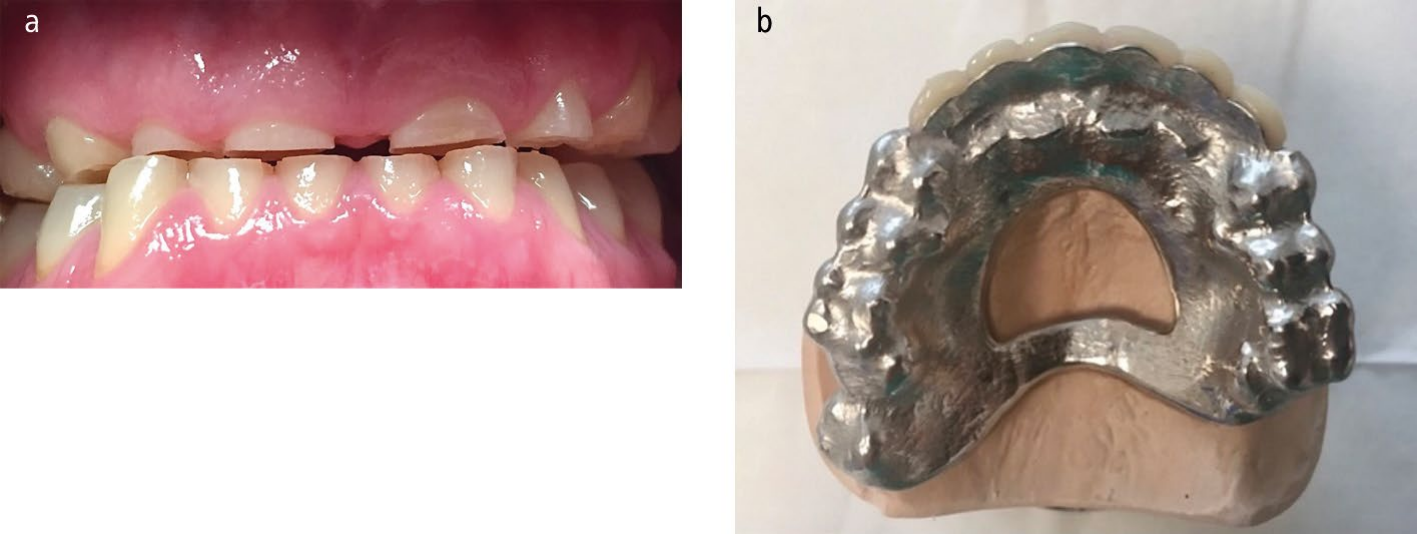
a, b) The bruxing patient from Figure 1 that had ground his maxillary teeth down almost to root level. He was rehabilitated with a maxillary partial chrome overdenture/overlay denture. Metal backings were prescribed to protect the anterior teeth and the posterior teeth were onlayed with metal to bring them back into occlusal contact. Metal was selected as it was less likely to fracture than acrylic in this hostile environment

In other cases, vertical height may not be reduced and the loss of tooth tissue is compensated for by eruption of the teeth and supporting tissues. In such cases, it may be necessary to increase the occlusal vertical dimension and then restore the entire arch to this new facial height in order to obtain adequate interocclusal space to restore the teeth. Such patients may have an intact (but worn) dentition and therefore tend to be treated with fixed restorations.^19^ Indeed, an increase in vertical dimension by 2-6 mm does not seem to cause any appreciable facial change in aesthetics.^20^^,^^21^ The fixed restorations can be direct placed composite resin or indirect intra- or extra-coronal restorations, with or without crown lengthening surgery. Crown Lengthening surgery and restoration of the teeth (without an increase in vertical dimension) will be required in cases where no increase in face height is needed." However, in some cases, the wear is so severe that only roots remain; such patients can benefit from being rehabilitated with a removable chrome-based overdenture/overlay denture at an increased OVD^21^ (Figures 8a and 8b).

In some cases, the increase in the OVD required to allow the provision of an overdenture or onlay is too great to allow reconstruction with an overdenture. As such, a profound increase in lower face height may look aesthetically incorrect. Such cases are more difficult to manage and may require extraction of a number of the remaining roots to allow for the alveolar process to resorb and remodel. The patient may then have to be rehabilitated with complete dentures or even an implant-supported superstructure. Both of these options are not ideal in such a hostile environment. Patients who brux on their complete dentures may complain of regular soreness and ulceration, while patients with implant-supported prostheses may have problems with superstructures or fixtures fracturing, screw loosening or loosening of their retentive components.^22^

## Restorative considerations when managing a bruxing patient

### Materials

No restorative material or technique is ideal to restore a patient who presents with destructive bruxism. However, composite resin is extremely useful in the reconstruction phase for these patients. Such restorations will fracture in this group of patients but are easily repaired chair side.

There are minimal biological complications associated with this treatment modality. Gulamali *et al*.^23^ reported that only 2% of their tooth wear cohort managed with composite resin needed intervention due to recurrent caries and patient satisfaction with composite restorations was high, despite the fact that 50% of their restorations had major failure. 'Heavy duty' metal ceramic crowns and other full coverage restorations may fail with catastrophic fracture, breaking the core of the tooth in an irretrievable way. The advantage of composite materials is therefore to act as stress breakers, preferentially failing to protect the remaining tooth tissue.

Therefore, a logical approach to rehabilitating worn anterior teeth in a parafunctional environment is to use direct composite resin. As a material, it has an acceptable survival probability in the short- to medium-term.^24^ Although the restorations may fail at an increased rate, relative to a non-bruxing patient, this still represents a quick, cheap, biologically friendly and repairable treatment modality, even when failure is likely. Kassardjian and colleagues^25^ have suggested that the annual intervention rate for direct composite restorations is 11.6%. Although this figure may seem relatively high, it still represents a purely additive and repairable mode of treatment.

**Fig. 9:**
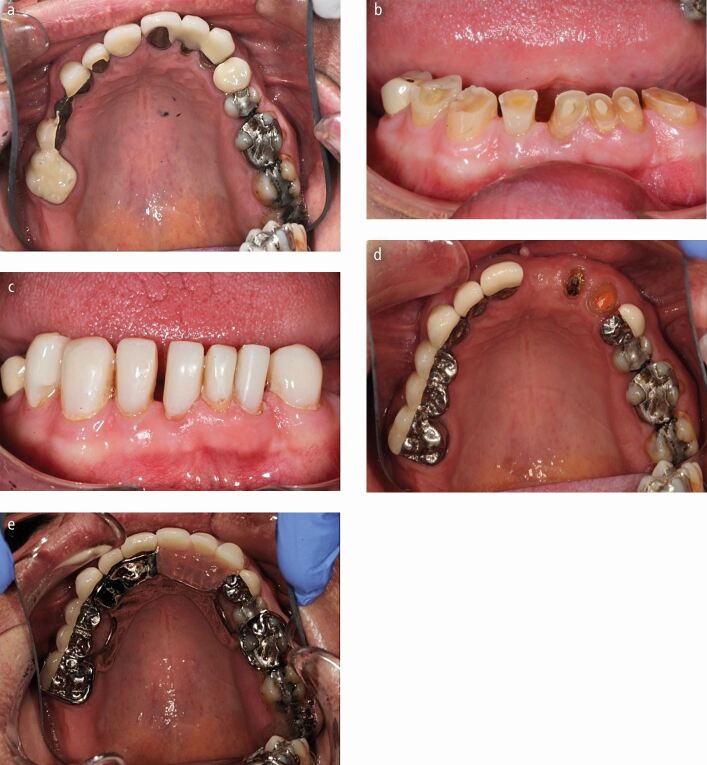
a, b, c, d, e) A bruxist presenting with failing crown and bridge work in the maxillary arch and a worn mandibular dentition. Notice how the worn lower teeth were restored with composite at an increased vertical dimension. The failing crowns and bridges in the maxilla were replaced with new castings and a partial chrome over denture. Notice the prescription of palatal and occlusal metal on the new castings. Note the retention of anterior roots to support the new overdenture. Ideally the new prosthesis should have had metal backings on the anterior teeth and a ring based major connector

Full coverage crowns may also be considered to rehabilitate such patients but they must be prescribed with caution and planned carefully. Any proposed increase in OVD can be trialled with direct composite resin initially, which can then act as the core for the extra-coronal, full coverage restorations. Clinicians should ensure that any metal ceramic crowns are designed with porcelain labially or buccally and metal palatally (for anterior crowns) and occlusally (for posterior teeth), where occlusal loads will be at a maximum (Figures 9a, 9b, 9c, 9d and 9e). In the posterior segment, an alternative to metal ceramic crowns could be full coverage gold crowns. Given the destructive nature of full coverage preparation in an already compromised tooth, the authors would recommend limiting this modality to the replacement of pre-existing, failing crowns in the dentition when possible.

### Onlays and overdentures

An alternative to full coverage restorations in the posterior region would be the use of indirect onlays. Partial coverage onlays can be used to restore any worn teeth with minimal preparation, will have supra-gingival margins for cleansability and will leave tooth structure exposed for future vitality testing should any symptoms occur after cementation. They would, therefore, seem preferable to full coverage crown preparation. A range of materials can be used, including composite, ceramic, gold and the newer hybrid ceramic/resin materials. Ceramic is probably best avoided in the posterior, load bearing region of a bruxing patient; given the likely increased fracture rate of restorations in this environment, ceramic will fracture and would be difficult to repair. Ceramics are also likely to cause enhanced wear of the opposing dentition. Suitable alternatives are tooth-coloured composite or gold alloy. Composite may have a higher rate of wear and failure but is financially cheaper and can be more easily repaired than ceramic. Gold alloy will have a lower rate of wear/fracture but is more costly to provide, given the cost of precious metal alloy and is highly dependent upon accurate fit. Chana *et al*.^26^ have reported an 89% survival rate of type III gold alloy onlays over a five-year period, suggesting that in the medium-term, they are a reasonable option to restore worn posterior teeth. The newer hybrid ceramic/resin materials are promising; materials like Enamic are etchable and can therefore be adhesively bonded on to an already worn tooth without significant preparation. It can also be repaired with composite resin after surface treatment with a silane coupling agent but there is very little long-term clinical data on such materials.

Removable overlays, onlays or overdentures may be realistic alternatives to rehabilitate severely worn dentitions, especially if the patient has long edentulous spans in addition to severely worn teeth and retained roots.^22^^,^^27^ The overdenture design has a significant role to play in many cases, in both partial and complete arch reconstruction. In a similar way to occlusal appliance failure described in paper 2, acrylic can fracture with the excessive loading placed upon it and therefore, a stronger, more rigid, chrome-cobalt framework should be prescribed. Any anterior prosthetic teeth should have metal backings for protection and to limit prosthetic failure.^27^ Posterior teeth should ideally be onlayed with metal rather than acrylic as it is less likely to fracture (Fig. 8b).

**Fig. 10:**
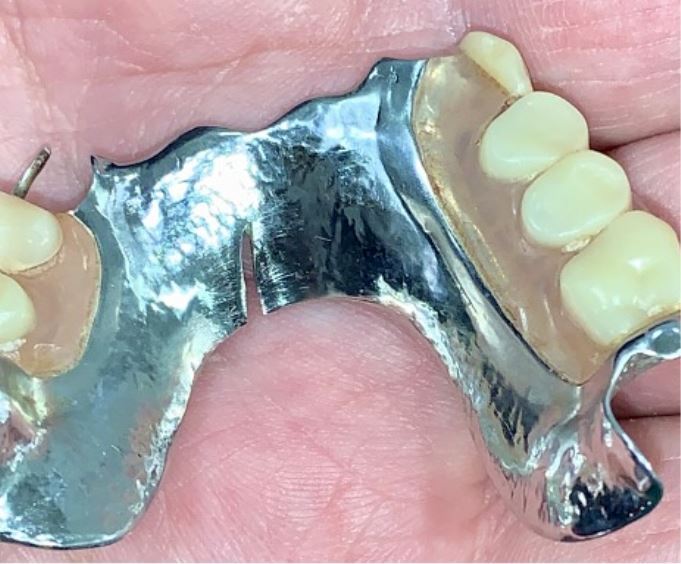
A U-shaped cobalt-chrome connector prescribed for a bruxist who managed to wear down his acrylic teeth and cause a fracture line to develop in his denture

To counter the loading and its consequent flexing of the denture baseplate across the arch, chrome-cobalt based ring connectors or plate connectors should be prescribed to increase support and retention for the prosthesis and may be required in either partial or complete denture designs. In the authors' experience, U-shaped connectors are not rigid enough in a bruxing patient and can flex or even fracture (Fig. 10).

**Fig. 11:**
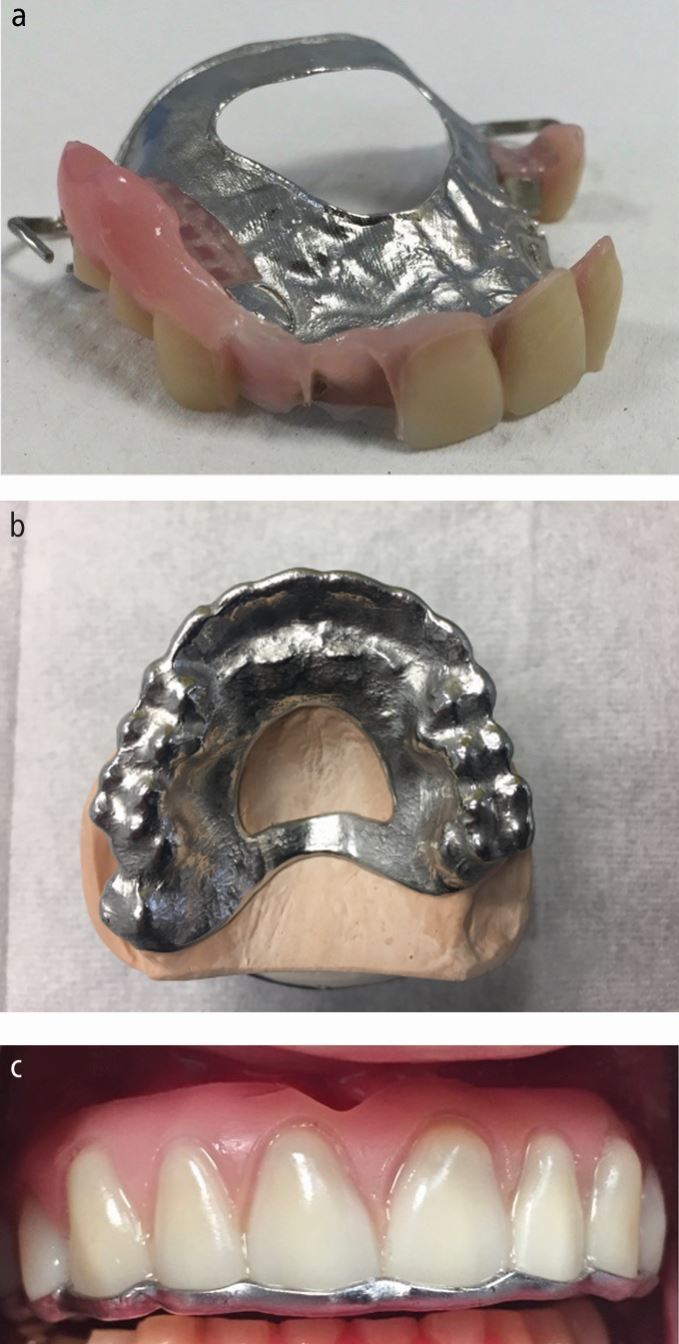
a, b, c) A bruxist with a protrusive habit. Despite having metal backings on his existing maxillary partial chrome denture, break off the acrylic teeth. A new chrome framework was made where there were metal and incisal onlays on the connector. The metal covering the incisal aspects of the anterior teeth prevented him from finding the acrylic/tooth interface and pushing the teeth off the framework

Some severely parafunctioning patients, particularly those with an exploratory type of bruxing behaviour, may be capable of moving their mandible through extreme lateral excursions. This may allow them to find the anterior acrylic tooth/metal interface of their overdenture. Over time, this can weaken and undermine the tooth/metal union. In these patients, it may be necessary to ensure that the metal backing 'lips over' the incisal edge of any anterior teeth to prevent further failure (Figures 11a, 11b and 11c).

### Retained roots

For patients with severe tooth wear, some teeth will have broken down to remain as retained roots. While roots may be unrestorable, they will provide support and proprioception, as well as inhibiting atrophy of alveolar bone and should be maintained if possible. Slavish removal of retained roots with little or no pathology is therefore not required but should symptoms arise, then extractions can be on an 'as needed' basis only.

### Implant-based reconstruction

For parafunctioning patients with missing units, prosthodontic replacement with dental implants can be difficult. Implants lack a periodontal ligament. They cannot provide the same level of proprioceptive feedback to a patient like a tooth-supported prosthesis. Implant fixtures are also rigid, with no ligament allowing physiological movement or resistance to loading. Therefore, a higher rate of prosthetic superstructure wear and fracture should be anticipated. The fixtures are also vulnerable to the impact of the occlusal loading and may fracture.

Systematic and retrospective reviews^28^^,^^29^^,^^30^ shows bruxing appears to increase the risk of failure of implant fixtures. Broadly, bruxing-related failure for implant-supported prostheses falls into two groups: biological and mechanical. Biological failure includes enhanced risk of peri-implantitis, marginal bone loss and bone remodelling, whereas mechanical failure includes screw/cement failures, porcelain fractures and fixture fracture. Inevitably, there is a lack of homogeneity in study design and even assessment criteria. It would appear, though, that in the main, a bruxing environment is more hostile to implants and their restorations.

It is therefore to be expected that cement-retained prostheses may loosen at a faster rate under the higher occlusal loads and implant screws could loosen or fracture at a higher rate in a bruxing patient, relative to a non-bruxing patient. Consequently, it may be prudent to consider alternative prosthodontic options in a bruxing patient, such as a removable chrome denture or adhesive bridge work. These options are less invasive and are simpler to manage should complications occur. If, however, these have been exhausted, then an implant-based reconstruction may have to be considered but careful planning of the occlusal loading must be incorporated into the design with maximum retrievability of components.

To facilitate this, screw-retained superstructures should be planned for. Should - or when - the abutment/bridge screw loosens, the screw holes will allow easy access for remedial care. Metal should be prescribed for the occlusal surfaces of any posterior prosthetic teeth. If a tooth-coloured option is requested, composite should be considered rather than porcelain, to allow for easier repairs when fracture of the prosthetic teeth is observed. If a fixed bridge or denture is provided, perhaps a spare should also be constructed. If the initial superstructure needs to be repaired, the patient could be provided with a spare superstructure while the primary prosthesis is being repaired. With more implant superstructures being digitally milled, patients may be provided with a digital copy of their implant work. If the patient then requires a new superstructure but has moved to a different area/registered with a new dental practitioner, a new superstructure can be more easily milled, avoiding any unnecessary delay.

If an implant-retained overdenture is provided where a bar or ball abutments have been prescribed, wear on the retaining components is likely to occur more rapidly than in non-bruxing patients and some clinicians have reported tooth wear occurring in the arch opposing the bar or ball abutment.^31^ Therefore, the provision of an occlusal splint to protect the remaining dentition while the overdenture has been removed (typically at night) seems logical.

### Long-term protection

Once the reconstruction has been completed, the provision of an occlusal appliance is likely to be advisable, to protect fixed restorations from damage, whether implant supported or not. While occlusal appliances may not control the bruxing, they will provide protection to the dentition and the restorations during night-time bruxing, by dispersing the loading across the entire arch and tooth structures, avoiding pressure points on the dentition that predispose to fracture.

## Conclusion

Bruxing is a complex, multifactorial issue. The drive to brux is centrally placed and it may be impossible to stop. The teeth and any associated restorations may be damaged as a result of the drive to brux. It is therefore important to consider the impact on the patient. This ranges from minimal to significant, with a negative quality of life impact. Bruxism will require management in some but not all cases and review of bruxism should be considered a dynamic process, with varying levels of diagnostic appraisal. Unfortunately, management strategies have a poor evidence base, but provision of occlusal splints for protection of the tissues seems logical and an assessment matrix has been described that can help rationalise clinical decision-making. For cases where tooth wear and/or tooth loss is significant, reconstruction of the severely damaged dentition is possible but is subject to a number of caveats and a significant maintenance burden. The authors hope that by sharing their experiences and failures in these papers, others may benefit and provide a better standard of care while managing their own bruxing patients.
